# Immune Responsiveness Induced by Administration of Heat‐Inactivated *M. bovis* and BCG Prior to Experimental Infection in African Buffalo

**DOI:** 10.1155/tbed/9279622

**Published:** 2026-10-17

**Authors:** J. M. Hewlett, L. M. de Klerk Lorist, E. P. Mitchell, L. J. Nupen, I. A. Sevilla, C. Gortazar, V. P. M. G. Rutten, R. Juste, A. L. Michel

**Affiliations:** ^1^ Department of Veterinary Tropical Diseases Faculty of Veterinary Science University of Pretoria Gauteng P/Bag X04 Pretoria 0110 South Africa up.ac.za; ^2^ Centre: Veterinary Wildlife Studies Faculty of Veterinary Science University of Pretoria Pretoria South Africa up.ac.za; ^3^ Department of Agriculture, Land Reform and Rural Development Skukuza South Africa; ^4^ Department of Paraclinical Sciences Faculty of Veterinary Science University of Pretoria Pretoria South Africa up.ac.za; ^5^ Department of Zoology and Entomology University of Pretoria Pretoria South Africa up.ac.za; ^6^ Department of Animal Health Neiker Derio Bizkaia Spain; ^7^ SaBio Instituto de Investigación en Recursos Cinegéticos (IREC) CSIC JCCM Universidad de Castilla La Mancha Ciudad Real Spain uclm.es; ^8^ Department Biomolecular Health Sciences Division Infectious Diseases and Immunology, Section Immunology Faculty of Veterinary Medicine Utrecht University Utrecht Netherlands uu.nl

**Keywords:** African buffalo, antibody response, BCG, bovine tuberculosis, heat-inactivated, immunisation, interferon-gamma release assay, intra-dermal tuberculin test, *Mycobacterium bovis*, *Syncerus caffer*

## Abstract

African buffalo (*Syncerus caffer*) are the primary wildlife reservoir for bovine tuberculosis (BTB) in Africa. Infected buffalo populations spread BTB to numerous species in conservation areas as well as to cattle at park boundaries. Quarantine requirements for BTB complicate wild animal metapopulation management. Considerable interest has arisen in the vaccination of cattle and indigenous wildlife species against BTB as an additional control strategy. This pilot trial aimed to evaluate cell‐mediated and humoral immune responses in African buffalo immunised against BTB. Twenty‐one sub‐adult African buffalo were divided into four treatment groups, and received either Bacillus Calmette–Guérin (BCG; 10^6^ CFU) intra‐muscularly, a heat‐inactivated *Mycobacterium bovis* vaccine intra‐muscularly (HIMB‐IM; 10^7^ CFUs; followed by a boost after 4 months) or orally (HIMB‐O; 10^7^–10^8^ CFU), or 1 mL phosphate‐buffered saline (PBS) intra‐muscularly (control group). 7 months after immunisation, all animals were intratracheally challenged with *M. bovis* (2 × 10^5^ CFU). Monthly Bovigam interferon‐gamma (IFNγ) assays and *M. bovis*‐specific ELISAs were conducted and in addition, comparative intra‐dermal tuberculin testings (CITTs) were performed at study start and termination 5 months after experimental infection. Over the study period, each treatment group showed distinct cell‐mediated responses which differed in strength and temporal dynamics. In the post‐immunisation stage, HIMB‐IM animals produced a strong increase in bovine–avian purified protein derivative (PPD) IFNγ release after booster immunisation while HIMB‐O and BCG groups showed relatively fewer numbers of positive responses. The experimental infection triggered significant elevations in bovine–avian PPD IFNγ release in all treatment groups. All animals were converted to bovine reactors. Both HIMB groups showed stronger IFNγ responses compared to the BCG and control groups in the early post‐infection period, suggesting superior immune priming. Antibody responses showed marked individual variation, episodic interference from non‐tuberculous mycobacterial (NTM) species and low test sensitivity and specificity.

## 1. Introduction

African buffalo (*Syncerus caffer*) are the most important wildlife maintenance hosts of *Mycobacterium bovis* in sub‐Saharan Africa [1]. Buffaloes disseminate bovine tuberculosis (BTB) principally through the pulmonary route and contribute to extensive ecosystem contamination with *M. bovis* [2, 3]. Kruger National Park (KNP), Hluhlwe–Imfolozi Park and Madikwe provincial parks together contain ~85% of South Africa’s buffalo population in conservation areas [4] and are all considered endemically infected with *M. bovis* [5]. At high prevalences, *M. bovis* infection reduces buffalo population growth rates through chronic, erosive effects on immune function, body condition, fecundity and drought tolerance [6–8], rather than via mass mortality. However, buffalo infect other wildlife maintenance hosts, multiple spillover host species, buffalo in transboundary conservation areas and cattle residing at park boundaries [9–12]. This constitutes a conservation concern, as spillover infections occur in a number of vulnerable or endangered species, such as wild dog (*Lycaon pictus*), cheetah (*Acinonyx jubatus*) and black rhino (*Diceros bicornis*) [13–15].

Test and slaughter (T&S) processes are impractical and disruptive to apply in large conservation areas [16]. Quarantine of infected conservation areas has stopped game sales and crucial metapopulation management procedures in several endangered species, including the white rhinoceros (*Cerathotherium simum*) [17]. Interest in immunisation against BTB in both wildlife and other species has grown considerably as an additional or alternative control measure to T&S strategies when the latter practice cannot completely control outbreaks, if an indigenous wildlife maintenance host is present or when implementation cost exceeds local resources [16, 18, 19]. Besides producing long‐lasting protective immune responses, an ideal immunisation for wildlife would require a single application, have low production costs and have a long shelf life without requiring a cold chain. Oral administration of vaccines in dangerous wild ungulates, such as African buffalo, would be cheaper and safer than via parenteral routes. Finally, a lack of adverse health effects in both target and non‐target species is important [20, 21].

Vaccination with Bacillus Calmette–Guérin (BCG) does not result in sterile immunity to BTB infection in cattle but has protective efficacy by reducing lesion severity and bacterial burden [22], which dramatically reduces the risk of transmission [23]. BCG is cheap to produce, safe and requires only a single intra‐dermal injection, but it induces positive intra‐dermal tuberculin skin test responses in cattle and thus requires differentiating infected and uninfected vaccinated animal testing [18]. Refrigeration is required during storage and transportation, which is difficult in resource‐constrained environments.

An *M. bovis* isolate from infected Eurasian wild boar (*Sus scrofa*) in Spain was used to produce a heat‐inactivated *M. bovis* (HIMB) vaccine. When administered orally or intra‐muscularly, it provides significant protection of *M. bovis* infection in wild boar [24, 25]. In European badgers, the oral formulation provides equivalent protection to that of oral BCG [26]. Orally vaccinated (uninfected) cattle did not show positive responses in comparative intra‐dermal tuberculin tests and interferon‐gamma (IFNγ) release assays [27]. In red deer, orally administered HIMB reduced the bacterial burden and significantly decreased lesion scores while not producing positive skin test results [28]. In domestic calves, subcutaneous administration produced significant cell‐mediated and humoral immune responses [29]. In addition, intra‐muscular HIMB in cattle has been shown to enhance phagocytosis in cattle through ‘training’ of the innate immune system, which aids in controlling bacterial load [30]. Besides not requiring refrigeration, an effective HIMB vaccine avoids the persistence of the live vaccine strain in vaccinated animals and the environment and potential adverse effects in non‐target animals [25, 31].

Numerous species of non‐tuberculous mycobacteria (NTM) are normally present on African buffalo [32]. Due to the presence of orthologous genes, hence similarity in epitopes, for example ESAT‐6 and CFP 10, of *M. tuberculosis* complex and particular NTM species [33, 34], exposure to NTMs can cause false positive results in ante mortem *M. bovis* tests [34–36] and may interfere with the development of protective immune responses to BCG in cattle [37, 38] and potentially in African buffalo following BCG immunisation [39].

The present study, part of a larger project investigating the applicability of HIMB vaccines to combat BTB, assesses their efficacy to elicit immune responsiveness in African buffalo and compares responsiveness with that in BCG‐vaccinated animals and controls. Effects of vaccination on BTB diagnostics were also investigated, as well as immune responses to specified NTM species.

## 2. Materials and Methods

This study was approved by the University of Pretoria Ethics and Research Ethics Committees (V036‐18; REC 148‐19), the South African National Parks Animal Use and Care Committee (MICA1589) and the Department of Agriculture, Land Reform and Rural Development, South Africa (Section 20 permit 12/11/1/1/6).

### 2.1. Study Animals

#### 2.1.1. Captures and Pre‐Movement Testing

Veterinary public health inspections of 150 adult buffalo carcases removed from the Manyoni reserve for meat processing occurred 6 months prior to study captures. Thinly sliced head and thoracic lymph nodes of all buffalo showed no granulomatous lesions (Hewlett, unpublished data). In addition, comparative intra‐dermal tuberculin testing (CITT) of buffalo removed sporadically from the Phinda reserve in previous years did not indicate *M. bovis* infection (Warren McCall, personal communication, September 2020).

Twenty‐four free‐ranging African buffalo (10 female, 14 male), aged 7–15 months, from the Phinda and Manyoni reserves, KwaZulu Natal, South Africa, were chemically immobilised remotely using thiafentanil 3–4 mg (Thianil, Wildlife Pharmaceuticals, Mpumalanga, South Africa) and azaperone 40 mg (Stresnil, 40 mg/mL, Janssen Pharmaceutical, Woodmead, South Africa) with a Pneu‐Dart 389 dart gun and tranquilised with haloperidol 10 mg (Kyron, Benrose, South Africa). Animals were microchipped, ear‐tagged and treated with a single subcutaneous dose (1 mL per 50 kg) of ivermectin (‘Ivomec’ Merial, Gauteng, South Africa). The animals were loaded into trucks, reversed with naltrexone at 10 times the thiafentanil dose (‘Trexonil’ Wildlife Pharmaceuticals, Rocky Drift, Mpumalanga, South Africa) and transported to on‐site holding facilities (bomas). Captures have been allocated the timepoint term ‘MA01’ (Figure 1).

**Figure 1:**
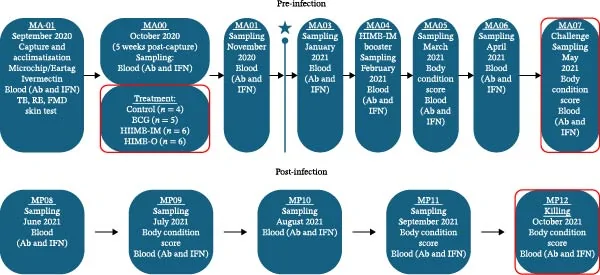
Monthly timeline indicating sampling, composite body condition scoring, testing, immunisation, treatments and *M. bovis* challenge from initial capture to euthanasia for 21 African buffalo ∗ indicates MA02, where no sampling occurred. MA, month ante; MP, month post‐infection.

To confirm that the study buffalo were free of BTB, the Bovigam IFNγ release assay and CITTs were performed at capture (see below). Serum samples were collected to perform pre‐movement testing for brucellosis [40] and foot‐and‐mouth disease [41]. Based on negative test outcomes, the calves were transported to the State Veterinary Quarantine Bomas, Skukuza, KNP.

#### 2.1.2. Housing and Husbandry at Study Site

Upon arrival at the study site, the 24 buffalo calves were divided randomly into two groups of 12 animals each, and each group was housed in two joint enclosures (each of 10 × 25 m) containing a night room and outside areas, which were partially shaded by netting and trees. They were fed *Eragrostis teff* and lucerne (2:1 ratio) twice daily and had ad lib access to water and mineral blocks. The feed was supplemented with game pellets (‘Barnlab’, Wildswinkel, Alpen Park, Pretoria). Animals were monitored daily for habitus, appetite, injury and bullying. From MA02, faecal floatation tests were performed monthly to quantify the levels of nematode and coccidia eggs in study animals. These results, together with body condition, habitus and stool quality, were used to determine when nematode and coccoidal infections were treated with either injectable doramectin (Dectomax, Zoetis, South Africa, 0.2 mg/kg) or water‐soluble amprolium (10 mg/kg, Kyron, Benrose, South Africa) throughout the study period.

### 2.2. Body Condition Scoring

Composite body condition scores of study animals were determined intermittently during the study period (MA05, MA07, MA09, MA11 and MA12) according to Ezenwa et al. [42]. While animals were immobilised, visual assessment and palpation of subcutaneous fat over ribs, dorsal hips, lumbar spine, dorsal vertebral bodies and tail base were performed using a score of 1–5 (1 being emaciated and 5 being very overweight; the sum of these five scores formed a composite score for each animal [42]). In addition, the chest girth was measured (cm) behind the front legs and used as an indication of the body size and weight [42]. Independent of the above measurements, qualitative visual assessment of body condition was performed weekly as part of animal husbandry.

### 2.3. Immunisation

After 5 weeks of acclimatisation, the buffaloes were immobilised and randomly allocated into three immunisation treatment groups and one control group (while remaining in their allocated enclosure groups). This time point was designated as ‘MA00’ (Figure 1). The control animals received 1 mL of phosphate‐buffered saline (PBS) in the left gluteobiceps muscle. The BCG group was given ‘BCG VACCINE SSI’ (Statens Serum Institut, Denmark) containing *M. bovis* Danish 1331 strain, 2–8 × 10^6^ CFU/0.1 mL reconstituted with diluted Sauton SSI shortly before vaccination. Subsequently, the content of one vial from the same batch was serially diluted and plated on Middlebrook 7H11 agar to obtain an accurate concentration of viable bacteria. Animals receiving BCG were moved to a third separate enclosure to prevent physical contact and possible transmission to other treatment groups and then received 1 × 10^6^ CFU in 1 mL in the left gluteobiceps muscle. The third group was immunised with a HIMB vaccine [24, 25] that was administered orally ‘HIMB‐O’ after mixing 2 mL of the oral vaccine formulation (1 × 10^7–8^ CFU/mL suspended in PBS) with bran and olive oil to form a paste, which was rubbed on the buccal mucosa. The final treatment group received 1 mL of the heat‐inactivated vaccine intra‐muscularly (‘HIMB‐IM’) into the left gluteobiceps muscle. It contained 1 × 10^6^ CFU and Montanide ISA 50 V (Seppic, Castres, France). A booster vaccination with HIMB‐IM was administered 4 months after the initial vaccination (MA04).

### 2.4. Immune Response Assessment

#### 2.4.1. Monthly Sample Collection and Processing

This study used CITT and IFNγ release assays and *M. bovis*‐specific antibody assays to represent cell‐mediated and humoral immune responses, respectively. During 11 monthly immobilisations, blood samples were collected from the jugular vein of each animal into serum and heparin tubes using a vacutainer system. Animals were not sampled at MA02 due to visually assessed poor body condition. Serum tubes were left for blood to clot overnight; after centrifugation (1050 ×*g* for 10 min), serum was harvested and frozen at −20°C until antibody assessment. Heparinised blood was processed within 6 h for IFNγ testing.

#### 2.4.2. Cell‐Mediated Responses: CITT

Mid‐cervical injection sites on the left and right sides of the neck were hand‐shaved, and the local skin fold thickness was measured with a Hauptner calliper. Then, intra‐dermal injection of 0.1 mL of bovine and avian tuberculin‐purified protein derivatives (PPDs) (PPD_BOV_, 3000 IU; PPD_AV_ 2500 IU, ThermoFisher Lelystad, Netherlands) was performed into the left and right sites, respectively, in accordance with the Veterinary Procedural Notice for Buffalo Disease Risk Management (https://old.dalrrd.gov.za/Branches/Agricultural-Production-Health-Food-Safety/Animal-Health/disease-control/buffalo).

The skin test responses were evaluated by visual inspection, gentle palpation and measurement of the skin fold thickness 72 h after tuberculin injection. A difference between the increases at the bovine and avian injection sites of ≥3 mm was used as a diagnostic cut‐off for a positive response. The presence of oedema or necrosis at the injection sites indicated a positive response, irrespective of skin fold thickness. If the difference between bovine and avian injection sites was <3 mm but the bovine site had an increase ≥3 mm, the animal was considered a bovine reactor and suspicious of having *M. bovis* infection [43], BTB manual, https://nahf.co.za/updated-tuberculosis-manual/).

#### 2.4.3. Bovigam (IFNγ) Release Assay

Within 6 h of sampling, 1.5 mL aliquots of heparinised whole blood were pipetted into 24‐well cell culture plates (CellstarGreiner Bio One) and stimulated with bovine and avian PPD (PPD_BOV,_ 900 IU; PPD_AV,_ 1500 IU) (ThermoFisher, Lelystad, Netherlands); a pokeweed mitogen as a positive control (at a final concentration of 5 μg/mL); and 100 μL Gibco RPMI 1640 medium (ThermoFisher Scientific) as a negative control. Stimulation with ESAT‐6, a recombinant *M. bovis* protein was done with 10 μg/mL blood. PPD prepared from *M. fortuitum*, *M. malmesburyense* and *M. nonchromogenicum*, as described by Jenkins et al. [44], was added on most test occasions at a final concentration of 330 μg/mL [44]. Following incubation at 37°C for 24 h, the supernatants were removed and assayed in the BOVIGAM (IFNγ) test kit for cattle (Prionics, Lelystad, Netherlands) as described by the manufacturer. In all assays, nil (negative control) optical density (OD) was subtracted from PPD and ESAT‐6 OD responses to correct for background responses. The interpretation of the test results was per Michel et al. [45] using OD >0.2 as a cut‐off for a positive result for bovine–avian PPD (IFN_γBOV–AV_), ESAT‐6 antigen and non‐tuberculous mycobacterial (NTM) species PPD IFNγ release assays [45].

#### 2.4.4. Humoral Immune Responses: *M. bovis* Specific Antibody Assay

Antibodies specific for *M. bovis* in buffalo serum were measured using a *M. bovis* antibody assay for cattle (IDEXX Laboratories Inc., USA) according to the manufacturer’s instructions with the modification described for buffalo using undiluted serum [46].

### 2.5. Experimental *M. bovis* Challenge

The *M. bovis* field isolate (SB0130) was collected from a buffalo showing macroscopic granulomatous lesions at post mortem that was propagated in Middlebrooks 7H9 medium at 37°C for 5 weeks. Bacteria were spun down, resuspended and homogenised to a single‐cell suspension in Middlebrook 7H9 medium and a dilution series was prepared using McFarland standard 0.5 as a turbidity reference. The bacterial concentration was adjusted microscopically to 5 × 10^5^ CFU/mL and verified at the time of preparing the challenge solution and again post‐infection by plating a serial dilution onto Middlebrook 7H11 agar plates (Merck, Johannesburg) and culturing at 37°C for 8 weeks. Thirty doses were frozen at −80°C until use. The final dose as determined post‐infection was 2 × 10^5^ CFU/2 mL.

Seven months (MA07) after initial immunisation, study animals were immobilised, sampled (including blood) and then experimentally infected with *M. bovis* (SB130). A mid‐ventral cervical area of skin (10 × 10 cm) was clipped and surgically prepared. An 18 G spinal needle (Spinocan, Braun, Germany) was inserted in between two tracheal rings, and the challenge dose in 2 mL PBS was inoculated into the tracheal lumen. The needle was rinsed with 1 mL of PBS. The incision wound was stapled, and the etorphine immobilisation was antagonised as before.

### 2.6. Immunisation Diagnostic Interference

To estimate immunisation interference in *Mycobacterium tuberculosis* complex (MTBC)‐specific cell‐mediated and humoral diagnostic tests, sensitivity and specificity were calculated in the pre‐infection and post‐infection stages. Given the persistent negative results of historical BTB testing in the herds of origin, which were confirmed in pre‐movement testing in the CITT and IFN‐γ release assays, samples obtained between immunisation and infection (pre‐infection: MA01 through MA07; Figure 1) were used as negative references for calculating the effect of vaccine immune response on test specificity. Intratracheal administration of the high‐dose *M. bovis* marked the start of the post‐infection period (MP08 through MP12; Figure 1); these samples were taken as a positive reference for estimating vaccine interference in test sensitivity [47].

### 2.7. Statistical Analysis

All response variables were tested for normality by visualising histograms and using a Shapiro–Wilk normality test in R software with the RStudio interface (Boston, Massachusetts; http://rstudio.com/). Normally distributed data were used in *t*‐tests or ANOVA and a post‐hoc Tukey HSD test to compare between and among groups. Non‐normal variables were compared between groups using the Mann–Whitney *U*‐test (two groups), Kruskal–Wallis rank sum tests and Dunn post‐hoc tests with a Holm adjustment (multiple groups, one category), or a Friedman test multiple comparisons (multiple groups, two categories). Tests with *p* values < 0.05 were considered statistically significant.

Composite body condition score and girth measurement were plotted in R (package ‘ggplot2’). Differences in CITT responses between treatment groups were investigated using a one‐way ANOVA and Tukey HSD test. Pre‐ and post‐infection results of bovine–avian PPD IFNγ release assays (IFN_γBOV–AV_) data were compared using Mann–Whitney *U*‐tests and a two‐sample Kolmogorov–Smirnov test. A Kruskal–Wallis multiple comparison test was then used to compare these data from each month in the pre‐infection and post‐infection periods. To look for differences in IFN_γBOV–AV_ release assay responses among the four treatment groups and between months, a Friedman test and Tukey HSD test were used. Treatment group IFN_γBOV–AV_ responses were also compared within months using ANOVA and post‐hoc Tukey HSD tests. Relationships between treatment groups and mean PPD_BOV–AV_, PPD_BOV_, ESAT‐6 and NTM IFNγ release assay ODs were visualised in RStudio.

Sample‐to‐positive (S/P) ratios of OD resulting from antibody assessment by ELISA were calculated as per the manufacturer’s instruction. The treatment group median S/P ratios were compared between pre‐ and post‐infection periods using a Mann–Whitney *U*‐test and a pairwise Wilcoxon test rank sum test with continuity correction and plotted with the ‘ggstatsplot’ package in R. Overall average S/P ratios, prevalence of S/P ratio positive animals and box plots by treatment group were plotted in R.

The IFN_γBOV–AV_ release assay responses were standardised by dividing each monthly control reading by the initial MA01 reading to identify which NTM antigen caused greater cross‐reactivity particularly in the period MA06–MA07. These standardised values were used for 2‐way ANOVA (by treatment group and month) for each antigen separately.

For pre‐infection positivity peaks, correlations were performed in Excel within treatment groups between monthly antibody and IFNγ release assay results and between CITT, antibody and IFNγ release responses for the time point MP12.

## 3. Results

### 3.1. Boma Adaption, Body Condition and Girth Measurement

Three animals died of enteric coccidia and Strongyle helminth infections in MA01, reducing the sample size to 21. To balance the treatment group size after mortalities, animal number 5 (initially assigned to the control group) was vaccinated orally with HIMB in MA04, and group composition in each boma was reshuffled to place animals of similar body size and temperament together. All animals lost body condition by 9 weeks in captivity but by MA05 had returned to normal condition. After experimental infection, chest girth consistently increased (Figure 2A), while composite body condition score peaked 2 months after infection (MP09; Figure 2B) then declined.

**Figure 2:**
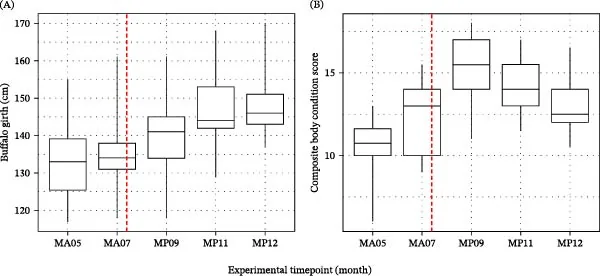
(A) Average African buffalo girth (cm) and (B) average composite body condition scores of all animals over the study period (experimental BTB infection indicated by dashed red line).

### 3.2. Comparative Intra‐Dermal Tuberculin Tests

The pre‐movement CITTs were negative for all animals (Table S1). At euthanasia (MP12), all animals were bovine reactors, and all animals had positive CITTs, except numbers 1 and 20, which were both classified as avian reactors and from the HIMB‐O group (Table 1). Central necrosis occurred in 52% of the animals, with those in the HIMB groups having the lowest incidence (Table 1). At MP12, differences in mean responses between treatment groups were not statistically significant, but control and HIMB‐O groups had the highest mean CITT responses (8.60 mm and 8.25 mm, respectively) and greatest variation (Table 1; Figure S1). Though not statistically significant, the HIMB‐IM group showed lower average skin swelling than the three other groups.

**Table 1:** Comparative intra‐dermal tuberculin test and bovine–avian interferon‐γ release assay results for 21 African buffalo at euthanasia, 5 months post‐experimental infection (MP12).

Buffalo number	Vaccine group	Comparative intra‐dermal tuberculin test (CITT)						Interferon‐γ assay (OD)	
		Δ Bovine PPD^a^	Δ Avian PPD	Δ BOV–AV^b^	Bovine injection site signs	CITT result^c^	Mean Δ BOV–AV	BOV–AV^d^	Mean BOV–AV
2	Control	17.2 (+)	2.1	15.1	Central necrosis	+	8.60	2.933	2.685
15		8.7 (+)	4.6	4.1		+		0.309	
21		7.8 (+)	5.4	2.4	Central necrosis	+		4.285	
26		21.2 (+)	8.4	12.8	Extensive necrosis	+		3.211	

3	BCG	6.6 (+)	−0.5	7.1		+	7.70	0.174	1.915
16		14.8 (+)	5.5	9.3	Central necrosis	+		3.502	
22		9.9 (+)	1.1	8.8	Central necrosis	+		2.345	
23		12.1 (+)	6.3	5.8	Central necrosis	+		0.898	
25		11.9 (+)	4.4	7.5		+		2.658	

4	HIMB‐IM	17.1 (+)	2.5	14.6		+	6.4	1.993	1.049
6		5.3 (+)	1.1	4.2	Central necrosis	+		0.09	
8		7.5 (+)	3.7	3.8	Central necrosis	+		0.951	
10		6.7 (+)	0.6	6.1		+		0.535	
14		7.2 (+)	0.8	6.4	Central necrosis	+		1.055	
17		3.9 (+)	0.8	3.1		+		1.668	

1	HIMB‐O	3.5 (+)	4.5	−1		N	8.25	0.39	1.712
5		19.9 (+)	3.9	16		+		1.342	
7		17.1 (+)	12.5	4.6	Central necrosis	+		1.334	
18		24.6 (+)	5.8	18.8	Extensive necrosis and oedema	+		1.84	
20		8.8 (+)	7.3	1.5		N		1.781	
24		11.6 (+)	2	9.6		+		3.583	

Abbreviation: N, negative test result.

^a^Δ Bovine PPD: Bovine reactor (+) designated when bovine site reaction is 3 mm or greater, irrespective of avian site response.

^b^Δ BOV–AV: Change in skinfold thickness elicited by Bovine PPD minus that elicited by Avian PPD (mm).

^c^CITT: A positive test ‘+’ is confirmed by a bovine site reaction 3 mm or greater than the avian site reaction or clinical signs including oedema and central necrosis at the injection site.

^d^IFNγ release assay: Positive response is PPD_BOV–AV_ OD ≥ 0.2.

### 3.3. IFNγ Release Assays

During the pre‐infection period (MA01–MA07), the buffaloes of the HIMB‐IM group showed positive IFNγ_BOV–AV_ responses at 64% of sampling timepoints, whereas HIMB‐O‐treated animals showed far fewer and less consistent positive responses (11%) (Table S2). The BCG and control groups showed the smaller number of positive responses during this period (0.3% and 0.9%, respectively) (Table S2). When comparing median IFNγ_BOV–AV_ responses in the pre‐ and post‐infection periods, significant differences were found for all animals (*W*_Mann–Whitney_ = 1473, *p* < 0.001) and across most months (*X* ^2^_Kruskal–Wallis_ = 153.22 *p* < 0.001). Median IFNγ_BOV–AV_ responses by treatment group also showed significantly higher post‐infection values (*p* < 0.001) in all groups (Figure S2). A two‐way ANOVA showed that the main contributor to the variance in IFNγ_BOV–AV_ responses was month of sampling (*F* = 24.04, *p* < 0.001), with lesser contributions from the treatment group (*F* = 3.83, *p* = 0.0106), and the interaction between these factors (*F* = 1.53, *p* < 0.05). When considering the entire post‐infection period, the control had significantly higher mean IFNγ_BOV–AV_ responses than the BCG group (*p* = 0.031, Figure S2).

When considering both monthly IFNγ_BOV–AV_ per treatment group (Figure 3A), the BCG and control groups remained negative prior to infection, while HIMB‐IM showed a strong, sustained positive IFNγ response, peaking in 2 months post‐vaccination and again 1 month after the booster vaccination. In MA07, HIMB‐IM showed a positive IFNγ response. Early in the post‐infection phase, the HIMB‐O and HIMB‐IM groups produced the strongest response (MP09), which then tapered off, whereas the control and BCG groups’ IFNγ release responses increased to the end of the study (MP12). Analysis of variance found mostly no significant differences between IFNγ_BOV–AV_ and the treatment group in the pre‐infection period. However, in the post‐infection period, the control group had significantly higher values than the BCG (*p* = 0.0473) and HIMB‐IM (*p* = 0.0198) groups in the MP11 and also the HIMB‐IM (*p* = 0.0003) and HIMB‐O (*p* = 0.0289) groups in the MP12 (Figure 3A). The bovine PPD (IFNγ_BOV_)‐stimulated blood showed response trends similar to those in IFNγ_BOV–AV_ assays; exceptions include the BCG group, which showed an early positive result (MA01) and all treatment groups tested positive in MA07, as opposed to only HIMB‐IM and HIMB‐O in the IFNγ_BOV–AV_ assays (Figure 3B).

**Figure 3:**
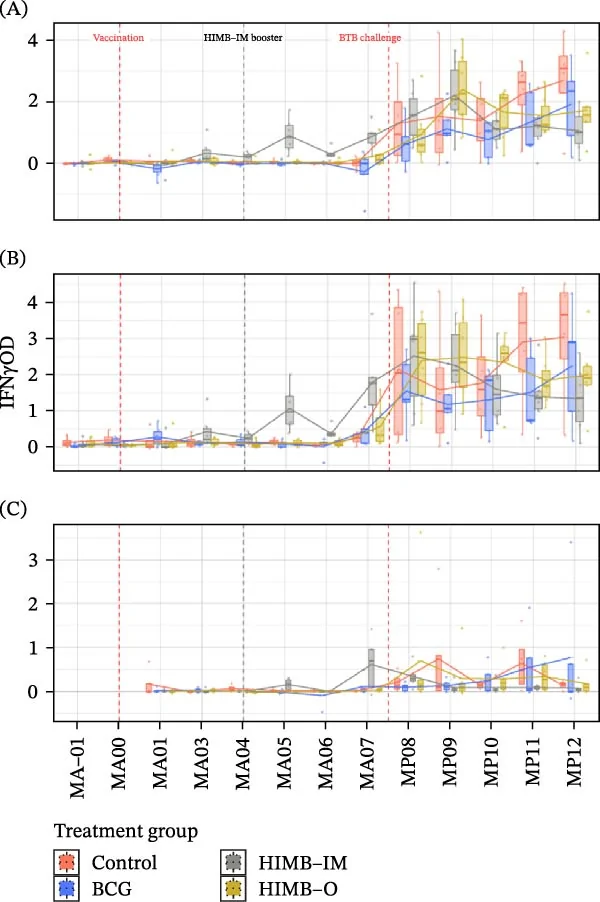
Monthly (A) bovine–avian PPD, (B) bovine PPD and (C) ESAT‐6 antigen IFNγ responses (OD) for each treatment group of African buffalo over the experimental period (lines connect means, dark crossbars are medians).

Overall, the ESAT‐6 antigen IFNγ (IFNγ_ESAT−6_) release responses (Figure 3C) were initially negative in the pre‐infection period (OD < 0.2). The HIMB‐IM group had an IFNγ_ESAT−6_ response peak in MA07; then, a sudden fall occurred, after which elevated response levels were maintained until MP12. The HIMB‐O group displayed a negative IFNγ_ESAT−6_ response before infection but reacted rapidly after infection and maintained elevated positivity until MP12. The control group exhibited peaks in values in MP09 and MP11 (Figure 3C).

### 3.4. NTM Elicited IFNγ Assays Responses Across Treatment Groups

IFNγ responses to *M. avium*, *M. fortuitum* and *M. nonchromogenicum* PPD (Figure 4) mostly remained low until MA07, when these NTM species’ PPDs elicited a large IFNγ response in all groups. This response coincided with elevated IFNγ PPD_BOV_ and IFNγ_ESAT−6_ responses in the HIMB‐IM group.

**Figure 4:**
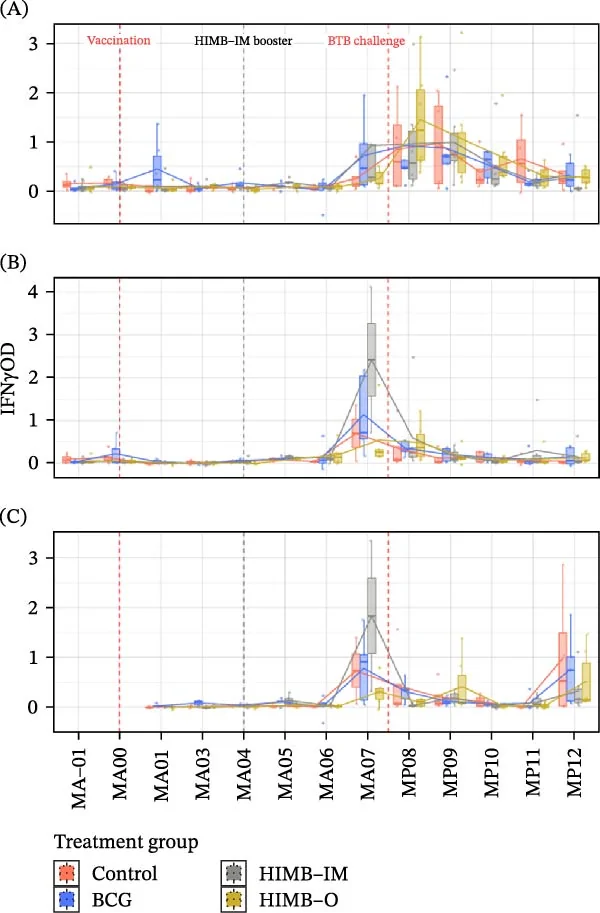
Monthly non‐tuberculous mycobacteria (A) *Mycobacterium avium*, (B) *Mycobacterium fortuitum* and (C) *Mycobacterium nonchromogenicum* PPD‐induced interferon‐γ values (optical density) for each treatment group of African buffalo over the experimental period (lines connect means, dark crossbars are medians).

The BCG‐treated animals had notable, enclosure‐specific *M. avium* PPD IFNγ responses in MA00 and MA01, respectively (Figure 4). *M. avium*, *nonchromogenicum* and *fortuitum* PPD‐induced multiple large IFNγ response peaks in MA07, with the HIMB‐IM and BCG groups having the largest responses. *M. avium* and *M. nonchromogenicum*‐induced response peaks also occurred after experimental infection. Response peaks in the *M. malmesburyense* PPD assays were in general of low amplitude (OD < 0.2), with only the HIMB‐IM and control groups showing positivity in MA05 and MP10, respectively.

When responses induced by NTM‐derived PPDs were compared to their initial value over the experimental period, the largest differences were observed in the *M. nonchromogenicum* PPD assay, in all immunisation groups at MA07, of which the highest was in the HIMB‐O (190‐fold the initial reading) (Figure 5). The control group reacted weakly to all NTM antigens prior to experimental infection but showed a strong increase to *M. nonchromogenicum* by MP12. This is in contrast with the stronger specific reactivity after *M. bovis* infection was seen in the control group, which was higher than that of all animals in the vaccinated groups (Figure 5)_._ The BCG group had the strongest specific response against PPD_BOV_ and the largest difference with PPD_AV_‐induced responses (Figure 5). The HIMB‐vaccinated groups had more non‐specific responses, especially against *M. nonchromogenicum* antigens with milder responses against the *M. bovis* antigens. The HIMB‐O group showed the strongest reactivity to *M. nonchromogenicum* and a mild response to the *M. bovis* antigen (Figure 5).

**Figure 5:**
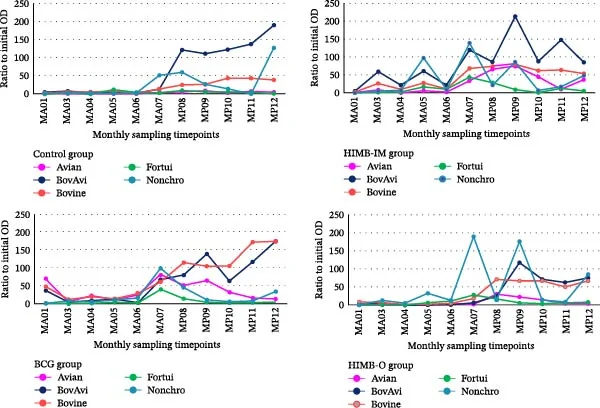
Interferon‐γ release assays for *M. bovis* (Bovine), *M. avium* (Avian), their difference (BovAvi), *M. fortuitum* (Fortui) and *M. nonchromogenicum* (Nonchro) PPD, as a ratio of initial values (MA01 not shown), for each treatment group of experimentally infected African buffalo (MA01–MP12).

### 3.5. *M. bovis* Specific Antibodies

Over the study period, control animals tested positive more frequently (43.8%) than immunised animals (HIMB‐O, 29.2%; HIMB‐IM and BCG both = 25.0%), and HIMB‐O had the largest mean S/P ratio (0.678), followed by the control (0.539) and HIMB‐IM (0.511), with the BCG group with the lowest value (0.290) (Table S3). While antibody responses did not differ significantly between treatment groups in the pre‐ and post‐infection periods (Figure S3), the proportion of animals testing positive trended from the lowest (<11.5%) during MA00–MA03 to the highest values of 66.6% and 52.4% in MP08 and MP10, respectively (Table S3).

Mean monthly antibody responses per treatment group showed bi‐modal patterns in all treatment groups (Figure 6). Marked individual variation in responses was noted, and the highest values occurred pre‐infection in MA07, in three animals with S/P ratios over 10: numbers 1, 5 (both HIMB‐O) and 4 (HIMB‐IM) (Table S3, Figure S3). The S/P ratio closely tracked the proportion of animals testing positive, except for MA07 (Figure S3). If MA07 is omitted, the correlation (*R*) between the S/P ratio and proportion of animals testing positive increases from 0.424 to 0.947.

**Figure 6:**
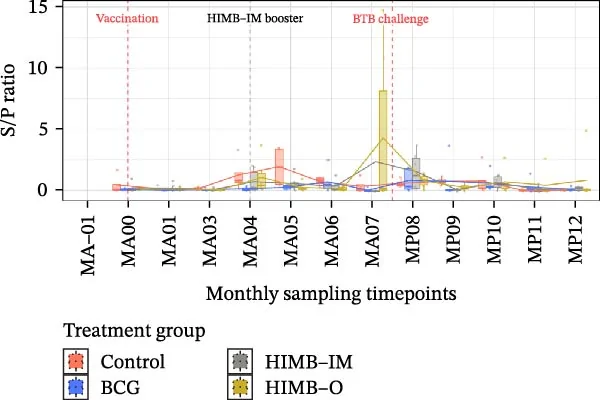
Monthly *M. bovis* antibody ELISA sample‐to‐positive ratios per treatment group (MA00–MP12) in experimentally infected African buffalo (lines connect means, dark cross bars are medians).

### 3.6. Immunisation Effect on Diagnostic Assays

Specificity calculated from the pre‐infection period was reduced in both the IFNγ release and antibody assay in all treatment groups, with BCG showing the least interference (Table 2). The lowest specificity was noted in the control group’s ELISA and the HIMB‐IM group’s IFNγ assay.

**Table 2:** Sensitivity and specificity of cell‐mediated and humoral tests performed on African buffalo during study period (test sensitivity and specificity from literature for African buffalo displayed below table).

Test	Treatment group	Specificity	Sensitivity		
			MP08	MP08–MP12	MP12
CITT	Control	100			100
	BCG	100			100
	HIMB‐IM	100			100
	HIMB‐O	100			66.7
IFNγ	Control	83.3	75.0	87.5	100
	BCG	91.1	80.0	80.0	80.0
	HIMB‐IM	46.3	100	91.7	83.0
	HIMB‐O	83.3	100	100	100
Antibody ELISA	Control	50.0	100	31.3	0.0
	BCG	77.5	20.0	25.0	20.0
	HIMB‐IM	72.9	0.0	20.8	0.0
	HIMB‐O	72.9	33.3	33.3	16.7

*Note:* CITT: Specificity 97%; sensitivity 76% [43]. Bovigam IFNγ ELISA assay: Specificity 92.6%; sensitivity 68.3% [45]. IDEXX *M. bovis* Antibody ELISA: Specificity 71.9%–94.7%; sensitivity 0%–54.6% [48].

In the control group, CITT sensitivity was good, and it improved with time in the IFNγ assay. The HIMB‐O group had reduced sensitivity of the CITT but good sensitivity to detect disease in early and later phases of infection on the IFNγ assay. With the BCG group, IFNγ assay sensitivity was initially lower than HIMB‐IM but remained constant, while sensitivity declined in the HIMB‐IM group. The ELISA sensitivity was high initially (MP08) in the control group but waned and, in general, was low in all treatment groups during the post‐infection period.

### 3.7. Correlations Between Test Results

Associations between the development of cell‐mediated and humoral immunity within treatment groups were investigated with correlation analysis to assess the possible effects of vaccination on the two immune responses. The BCG group had moderate to good correlations between *M. bovis* antibody responses and IFNγ_BOV_ (*R* = 0.650) and IFNγ_BOV–AV_ (*R* = 0.894) and IFNγ_BOV–AV_ and CITT reactions (*R* = 0.774). Correlations between these variables in the other groups were low, and the control group had a strong negative (*R* = 0.980) correlation between IFNγ_BOV_ and antibody responses.

Correlations were also used to investigate relationships between pre‐challenge peaks in antibody titres and mycobacterial IFNγ assays. In the control group, the MA05 antibody ELISA peak had moderate correlation with *M. nonchromogenicum* IFNγ assay (*R* = 0.689), *M. avium* (*R* = 0.644) and *M. malmesburyense* (*R* = 0.549), and poor correlation with IFNγ assay for *M. bovis* (*R* = 0.162). The MA06 peak in the BCG mean antibody response had a strong correlation with *M. fortuitum* IFNγ (*R* = 0.918). Despite this poor correlation between cell‐mediated and humoral immunity tests, the HIMB‐IM and HIMB‐O groups show positive mean IFNγ_BOV–AV_ responses and antibody peaks prior to infection (MA07), which coincides with positive peaks in *M. avium*, *M. fortuitum* and *M. nonchromogenicum* PPD IFNγ assays. The large HIMB‐IM group antibody peak in MA07 had poor correlation with IFNγ_BOV_ (*R* = 0.055) and IFNγ_AV_ (*R* = 0.045) but moderate correlation with the IFNγ_ESAT−6_ assay (*R* = 0.554).

## 4. Discussion

Over the 12‐month study period, each treatment group of African buffalo showed distinct cell‐mediated immune responses differing in strength and temporal dynamics. Pre‐infection, the HIMB‐IM immunisation elicited strong immune responsiveness (IFNγ_BOV–AV_), while other groups, especially the BCG group, showed fewer positive responses during this period. Production of IFNγ by T helper 1 lymphocytes is a critical factor inhibiting *M. bovis* replication and spread [22, 49, 50]. Whereas IFNγ is not considered a direct correlate of protection [51], its production indicates that the HIMB‐IM immunisation stimulates immune development against *M. bovis* antigens.

In the early post‐infection period (MP08 and MP09), both the HIMB‐IM and HIMB‐O groups showed rapid antigen‐specific reactivity, as reflected by the stronger IFNγ_BOV–AV_ production and the foremost IFNγ_ESAT−6_ peaks (Figure 4), relative to the control and BCG groups. Similar dynamics were noted in cattle, coinciding with *M. bovis* proliferation phases [52] and attributed to strong specific cell‐mediated reactions to low‐weight, early secretory proteins, in particular ESAT‐6. This early‐priming effect is followed by later immune responsiveness to a ‘wider range of antigens’ [53]. Compared to BCG, which lacks the ESAT‐6 gene [54], the presence of ESAT‐6 and other pathogen‐derived antigens in HIMB may contribute to rapid, accentuated post‐infection cell‐mediated responses. Prompt, strong IFNγ_ESAT−6_ responses in cattle have been noted in other immunisation studies and were associated with reduced BTB disease severity and protection [22, 55, 56].

In the later stages of infection, HIMB groups had lower cell‐mediated responses, also observed in cattle and associated with protection. In the late post‐infection stages (MP10–MP12), cell‐mediated responses (IFNγ_BOV–AV_) in the control group were significantly higher than those in immunised groups. In addition, relative to the HIMB groups, the BCG group had a later onset of IFNγ_ESAT−6_ responses (MP09 and MP11). In infected cattle, higher IFNγ_ESAT−6_ responses at intermediate post‐infection periods (4–5 months) were associated with increased lesion severity and a higher bacterial burden [22]. Sustained elevations in IFNγ responses in later stages of infection have been associated with greater bacterial load in cattle [57] and worsening disease severity [51, 58, 59]. It is unknown whether this association is also true for buffalo and will still be investigated for our study animals. Similar cell‐mediated immune dynamics were also reflected in CITT results, where control animals exhibited the largest reactions, which in that stage again suggests reduced control of *M. bovis* infection [60]. The IFNγ_ESAT−6_ responses in the control group peaked at 8 weeks post‐infection and then broadly oscillated, which could indicate fast specific changes in the rhythm of BTB lesion progression [61]. This group also showed the greatest variation in overall post‐infection IFNγ_BOV–AV_ and CITT responses, most likely reflecting individual differences in disease progression [62]. The smaller size and reduced variation in CITT responses in the intra‐muscularly immunised animals suggest that this route of administration had the most consistent regulation of delayed hypersensitivity reactions. In the HIMB‐IM group, positive IFNγ_BOV–AV_ assays and antibody responses in the pre‐infection period indicated notable responsiveness to immunisation, but also reduced test specificity and an elevated likelihood of positive CITT, IFNγ and ELISA responses in vaccinated, but *M. bovis* uninfected, African buffalo.

Immunisations administered to buccal membranes can induce immune responses locally at oral, nasal and respiratory mucosa and systemically [63, 64] and thus have the benefit of producing protective immune responses at sites of aerosol *M. bovis* infection [65]. Overall, the HIMB‐O group rarely caused cell‐mediated responses post‐immunisation, but it produced rapid, strong and specific post‐infection responses. The reduced impact on specificity noted with oral HIMB immunisation has also been observed in cattle [27]. Potentially protective cell‐mediated responses and the lower cost of oral vaccine application make this treatment a good candidate vaccine for the immunisation of free‐ranging African buffalo.

Although previously undervalued for intracellular pathogens, humoral responses following immunisation (BCG and HIMB) have been shown to contribute to protection against *M. tuberculosis* complex infection [25, 66, 67]. Although antibody responses were not significantly different between pre‐ and post‐ infection periods nor between treatment groups, immunised animals produced lower and fewer antibody responses than control animals. Response levels in this study were comparable to those found in *M. bovis* free‐ranging infected African buffalo [46]. The antibody responses in the present study may reflect poor reactivity between the anti‐bovine conjugate used in the assay and African buffalo IgG, resulting in a reduction in test sensitivity [68]. In contrast, parenteral HIMB immunisations have induced significant humoral responses in domestic calves [29], wild boar [24, 25] and rabbits [69]. The low pre‐infection humoral responses in the BCG group may have been a consequence of the low production of the MPB70 antigen in Danish BCG [70, 71]. Variable levels of antibody responses to MPB83 and MPB70 following BCG vaccination have been reported in cattle, ranging from low levels to those significantly different from those of unvaccinated cattle [72]. In addition to putative low test responsiveness, high individual variability and NTM‐induced cross‐reactivity may have contributed to this test being sub‐optimal for assessing the development of humoral immune responsiveness subsequent to BCG and HIMB immunisation in African buffalo.

False‐positive results on ante mortem *M. bovis* tests are known to occur following exposure to NTM species in cattle and African buffalo [35, 36, 44, 73]. In this study, in vitro cell‐mediated immune responsiveness elicited by NTM species PPDs often correlated or coincided with pre‐infection positive cell‐mediated and humoral responses in all treatment groups. In the pre‐infection period, the control group showed an antibody peak which had moderate correlation with IFNγ responses of three different NTMS (correlation coefficients range between 0.549 and 0.689), which may have contributed to reduced test specificity in this group. The BCG group had an *M. avium* PPD IFNγ response in MA01 and then an antibody peak in MA06, which may have been due to immunisation, but which also had good correlation with the *M. fortuitum* PPD IFNγ assay. Pre‐immunisation exposure to *M. avium* has been shown to significantly reduce or completely inhibit BCG replication in mice, preventing the induction of an effective immune response [37]. Mice exposed to oral *M. avium* prior to BCG immunisation exhibited decreased splenic lymphocyte IFNγ responses and switched to type 2 immunity [74]. Furthermore, it is suspected that NTMs contributed to the poor response to BCG immunisation in African buffalo in a previous study [39]. Both HIMB groups, especially HIMB‐O, showed pre‐infection cell‐mediated and humoral responses that coincided with NTM responses, particularly with *M. avium* and *M. nonchromogenicum*. This was also reflected in the final CITT, as the HIMB‐O group had two avian reactors and thus reduced sensitivity on the final CITT. As large numbers of NTM species have been isolated from soil, water troughs and nasal and pharyngeal swabs from captive African buffalo [32, 33], it is expected that orally immunised animals would show the greatest frequency and amplitude of responses to NTMS. In this study, NTM effects appeared to be influenced by NTM species and treatment group, with HIMB‐O animals showing the greatest cross‐reactivity. Further investigations are required to understand immune priming and cross reactions with NTMs [75] and their effect on immunisation efficacy.

Yearling African buffalo were sourced from herds with a longstanding BTB‐free status, confirmed by recent surveillance activities and negative CITT results upon capture. Infrastructure limitations prevented the replacement of study animals that died of parasite infection. To ensure *M. bovis* infection in all study animals, a high infection dose of a strain with known pathogenicity was used. The success of experimental infection with *M. bovis* was confirmed by significant elevations of IFNγ_BOV–AV_ subsequent to infection and the conversion of all study animals to, at least, bovine reactors. Reduced body condition was the main clinical sign of infection, which became evident 2 months post‐infection, as also noted in naturally infected free‐ranging African buffalo [6].

Vaccination of domestic calves with BCG results in optimal protective immune responses when administered between birth and 6 weeks of age [76]. This is due to the reduced risk of NTM exposure and relatively high systemic levels of natural killer (NK) cells. Dendritic cells that phagocytised BCG express Th1 cytokines, which activate this large pool of NK cells, which then produce IFNγ and modulate adaptive immunity in very young animals [77]. Wild animals experience high levels of stress when in captivity [78]. Weaned buffalo, rather than those at the optimal age for vaccination, were selected for this study primarily to avoid maladaptation to captivity and mortality. In addition, financial and housing constraints restricted the sample numbers and the size of animals used in this study. The small study size in combination with marked individual variation noted in African buffalo complicated the analysis of data. However, multiple studies evaluating BTB immunisation effect have been able to show significant differences between treatment groups in cattle and European badger using sample numbers similar to those used in this study [22, 29, 79, 80].

## 5. Conclusion

This pilot study aimed to investigate cell‐mediated and humoral immunity responses induced by HIMB administered intra‐muscularly, respectively, orally to African buffalo, followed by experimental infection. The HIMB‐IM immunisation elicited strong cell‐mediated responses pre‐infection. In the post‐infection period, compared to control and BCG groups, both HIMB‐IM and HIMB‐O produced *M. bovis*‐specific (ESAT‐6) cell‐mediated responses, potentially associated with reduced BTB disease severity. While cell‐mediated responses to HIMB immunisations are suggestive of a protective response, further pathological and microbiological investigations are required to determine whether the immune responsiveness observed is associated with protection. These will be dealt with in successive reports on the bacterial burden and number and severity of lung and lymph node lesions.

## Author Contributions

**A. L. Michel**, **V. P. M. G. Rutten and R. Juste**: conceptualisation. R. Juste, **I. A. Sevilla and C. Gortazar**: heat‐inactivated *M. bovis* formulation. **A. L. Michel**, **J. M. Hewlett and L. M. de Klerk Lorist**: permissions, planning, methodology, data collection. **R. Juste and L. J. Nupen**: data analysis and figures. **J. M. Hewlett**: writing. **E. P. Mitchell**, **V. P. M. G. Rutten**, **R. Juste**, **A. L. Michel and C. Gortazar**: review and editing.

## Funding

This research was supported by the Belgian Directorate‐General for Development Cooperation (DGD) within the DGD‐ITM Framework Agreement 4, AfriVet Bimeda, AgriSETA, the University of Pretoria and the Wildlife Group of the South African Veterinary Association.

## Disclosure

All authors have approved the final version of the manuscript. The materials and methods of this study were presented at Faculty Day August, 2023, Faculty of Veterinary Science, University of Pretoria (https://repository.up.ac.za/bitstream/handle/2263/93240/Faculty-Day-2023-A4-booklet-297x210mm-ONLINE-190923.pdf?isAllowed=y&sequence=1).

## Conflicts of Interest

The authors declare no conflicts of interest.

## Supporting Information

Additional supporting information can be found online in the Supporting Information section.

## Supporting information


**Supporting Information** Table S1: Comparative intra‐dermal tuberculin test results for 21 African buffalo at initial captures (MA01). Figure S1: Comparative intra‐dermal tuberculin test Δ bovine–avian PPD responses for 21 African buffalo at euthanasia (MP12). Dashed line: mean; solid line: median; error bars: standard deviation. Figure S2: Bovine–avian Interferon‐γ release assay results per treatment group during the pre‐infection period (MA00–MA07) and post‐experimental infection period (MP08–MP12) compared in African buffalo (solid lines represent medians;  ^∗^Indicates significant difference at *p* < 0.05). Table S2: Bovine–avian interferon‐γ release assay responses for 21 African buffalo over the study period. Table S3: IDEXX M. bovis antibody ELISA sensitivity to positivity ratios for 21 African buffalo over the study period. Figure S3: Comparison of sample‐to‐positive ratios (S/P) per treatment group, for pre‐infection period (MA00–MA07) and post‐infection (MP08–A12) periods in 21 African buffalo vaccinated against Mycobacterium bovis (solid lines represent medians).

## Data Availability

All data generated or analysed during this study are included in this published article and its Supporting Information.
