# Supplementary material for: Immune Responsiveness Induced by Administration of Heat‐Inactivated M. bovis and BCG Prior to Experimental Infection in African Buffalo
Source: Transbound Emerg Dis. 2026 Oct 17;2026:9279622. doi: 10.1155/tbed/9279622 (PMC13675938; doi:10.1155/tbed/9279622)
Supplement: Supplementary file 1 [file TBED-2026-9279622-s001.docx]

**Immune responsiveness induced by administration of heat inactivated *M. bovis* and BCG prior to experimental infection in African buffalo**

**SUPPLEMENTARY MATERIALS**

Table S1: Comparative intradermal tuberculin test results for 21 African buffalo at initial captures (MA-01).

| **Buffalo number** | **Vaccine group** | **Comparative intradermal tuberculin test** | | | | |
| --- | --- | --- | --- | --- | --- | --- |
|  |  | Δ Bovine PPD * | Δ Avian PPD | Δ BOV-AV ** | **Bovine injection site signs** | **Result**  ******* |
| 2 | CONTROL | -1,0 | -1.5 | 0.5 | None | N |
| 15 |  | 0,3 | -0,4 | -0,1 | None | N |
| 21 |  | -0,1 | -1,5 | 1,4 | None | N |
| 26 |  | -0,5 | -0,4 | -0,1 | None | N |
| 3 | BCG | -0,7 | -1,8 | 1,1 | None | N |
| 16 |  | -1,6 | -0,1 | -1,5 | None | N |
| 22 |  | 0,1 | -0,5 | 0,6 | None | N |
| 23 |  | 0,1 | 1,0 | -0,9 | None | N |
| 25 |  | 0,9 | 0,7 | 0,2 | None | N |
| 4 | HIMB-IM | -0,4 | 0,4 | 0,0 | None | N |
| 6 |  | 0,1 | -1,1 | 1,2 | None | N |
| 8 |  | -1,3 | -0,4 | -0,9 | None | N |
| 10 |  | -2,0 | -0,9 | -1,1 | None | N |
| 14 |  | 1,7 | -0,4 | 2,1 | None | N |
| 17 |  | -3,0 | -0,5 | -0,5 | None | N |
| 1 | HIMB-O | -0.7 | -0,4 | -0,3 | None | N |
| 5 |  | -0,7 | -2,6 | 1,9 | None | N |
| 7 |  | -1,6 | -1,6 | 0,0 | None | N |
| 18 |  | -1,9 | -3.3 | 1,4 | None | N |
| 20 |  | 2,0 | 2,2 | -0,2 | None | N |
| 24 |  | -0,1 | -0,7 | 0,6 | None | N |

*Δ Bovine PPD: Bovine reactor (+) designated when bovine site reaction is 3 mm or greater, irrespective of avian site response

**Δ BOV-AV: Change in skinfold thickness elicited by Bovine PPD minus that elicited by Avian PPD (mm)

***CITT: A positive test ‘+’ is confirmed by a bovine site reaction 3 mm or greater than the avian site reaction or clinical signs including oedema and central necrosis at the injection site. N: negative test result.

Figure S1: Comparative intradermal tuberculin test Δ bovine-avian PPD responses for 21 African buffalo at euthanasia (MP12). Dashed line: mean; solid line: median; error bars: standard deviation.

Figure S2: Bovine - avian Interferon ɣ release assay results per treatment group during pre-infection period (MA00 – MA07) and post- experimental infection period (MP08 - MP12) compared in African buffalo (solid lines represent medians; * indicates significant difference at p < 0.05).

Table S2: Bovine - avian interferon ɣ release assay responses for 21 African buffalo over the study period.

| **Buffalo identity** | **Vaccine treatment group** | **SAMPLING MONTH** | | | | | | | | | | | | |
| --- | --- | --- | --- | --- | --- | --- | --- | --- | --- | --- | --- | --- | --- | --- |
|  |  | **MA-01** | **MA00** | **MA01** | **MA03** | **MA04** | **MA05** | **MA06** | **MA07** | **MP08** | **MP09** | **MP10** | **MP11** | **MP12** |
| 2 | **CONTROL** | -0.041 | 0.02 | 0.019 | 0.039 | 0.034 | -0.027 | -0.051 | 0.036 | 0.03 | -0.049 | 0.009 | 3327.0 | 2.933 |
| 15 |  | -0.007 | 0.148 | -0.009 | 0.097 | -0.043 | 0.044 | 0.012 | 0.230 | 0.310 | 0.476 | 0.969 | 0.375 | 0.309 |
| 21 |  | -0.009 | 0.033 | 0.015 | 0.023 | -0.013 | 0.147 | 0.016 | -0.011 | 3246.0 | 1387.0 | 1928.0 | 2408.0 | 4285.0 |
| 26 |  | -0.019 | 0.235 | 0.235 | 0.157 | 0.1093 | 0.033 | -0.013 | -0.292 | 1564.0 | 4246.0 | 2618.0 | 2.85 | 3211.0 |
| MEAN | | -0.019 | 0.109 | 0.065 | 0.079 | 0.022 | 0.049 | -0.009 | -0.009 | 1202.585 | 1408.357 | 1136.744 | 1434.556 | 1874.811 |
| MEDIAN | | -0.014 | 0.091 | 0.017 | 0.068 | 0.011 | 0.039 | -0.001 | 0.013 | 782.155 | 693.738 | 964.485 | 1205.425 | 1606.967 |
| 3 | **BCG** | -0.005 | 0.026 | 0.0 | 0.014 | -0.101 | -0.069 | 0.024 | -0.360 | 0.067 | 0.022 | -0.015 | 0.596 | 0.174 |
| 16 |  | -0.078 | 0.037 | -0.055 | 0.03 | 0.023 | 0.003 | 0.009 | -0.008 | 1789.0 | 0.972 | 1319.0 | 2.3 | 3502.0 |
| 22 |  | -0.014 | 0.097 | -0.643 | 0.063 | -0.042 | -0.074 | 0.058 | 0.372 | 0.620 | 1401.0 | 1044.0 | 2579.0 | 2345.0 |
| 23 |  | 0.003 | -0.067 | -0.367 | 0.063 | 0.036 | 0.128 | -0.012 | 0.136 | 0.862 | 0.902 | 0.186 | 0.547 | 0.898 |
| 25 |  | -0.008 | 0.075 | 0.050 | -0.007 | -0.051 | -0.002 | -0.051 | -1.560 | -0.276 | 2253.0 | 1312.0 | 0.593 | 2658.0 |
| MEAN | | -0.020 | 0.034 | -0.203 | 0.033 | -0.027 | -0.003 | 0.006 | -0.284 | 358.055 | 731.179 | 735.034 | 516.607 | 1701.214 |
| MEDIAN | | -0.008 | 0.037 | -0.055 | 0.030 | -0.042 | -0.002 | 0.009 | -0.008 | 0.620 | 0.972 | 1044.0 | 0.596 | 2345 |
| 4 | **HIMB-IM** | 0.055 | 0.034 | -0.012 | 0.064 | 0.001 | 1068 | 0.649 | 2.08 | 2703 | 3287 | 0.878 | 1155.0 | 1993 |
| 6 |  | -0.02 | 0.051 | 0.049 | 0.064 | 0.391 | 1727 | 0.32 | 0.973 | 0.493 | 0.446 | 0.498 | 0.788 | 0.090 |
| 8 |  | 0.004 | 0.056 | -0.016 | 0.502 | 0.187 | 0.449 | 0.228 | 0.969 | 0.7 | 1.670 | 0.743 | 1315.0 | 0.951 |
| 10 |  | 0.063 | 0.01 | -0.17 | 0.217 | 0.221 | 1274.0 | 0.267 | 0.654 | 2263.0 | 1942.0 | 1659.0 | -0.003 | 0.535 |
| 14 |  | 0.005 | -0.002 | 0.019 | 1086 | 0.295 | 0.62 | 0.233 | 1497.0 | 1549.0 | 3665.0 | 1356.0 | 2442.0 | 1055.0 |
| 17 |  | -0.114 | -0.01 | 0.063 | 0.034 | 0.033 | 0.226 | 0.077 | 0.226 | 1574.0 | 2202.0 | 1379.0 | 1512.0 | 1668.0 |
| MEAN | | -0.001 | 0.023 | -0.011 | 181.147 | 0.188 | 678.387 | 0.296 | 250.317 | 1348.366 | 1849.686 | 732.687 | 1070.798 | 786.263 |
| MEDIAN | | 0.0045 | 0.022 | 0.004 | 0.1405 | 0.204 | 534.310 | 0.250 | 0.971 | 1561.5 | 2072.0 | 678.439 | 1235.0 | 527.976 |
| 1 | **HIMB-O** | 0.033 | 0.034 | -0.001 | 0.185 | 0.040 | -0.001 | 0.063 | 0.303 | 0.291 | 0.824 | 0.233 | 1236.0 | 0.390 |
| 5 |  | -0.212 | 0.001 | -0.020 | 0.181 | 0.233 | 0.115 | 0.175 | 1084.0 | 2829.0 | 3433.0 | 2159.0 | 2644.0 | 1342.0 |
| 7 |  | -0.012 | 0.070 | -0.008 | -0.039 | 0.004 | 0.004 | -0.044 | 0.058 | 0.596 | 4033.0 | 0.852 | 1025.0 | 1334.0 |
| 18 |  | 0.015 | 0.003 | 0.028 | 0.047 | -0.002 | -0.015 | 0.008 | 0.164 | 0.578 | 1566.0 | 2065.0 | 2047.0 | 1.840 |
| 20 |  | -0.016 | 0 | 0.012 | -0.003 | 0.021 | 0.088 | -0.061 | -0.045 | 0.031 | 2922.0 | 2362.0 | 1258.0 | 1781.0 |
| 24 |  | -0.040 | -0.064 | 0.425 | -0.265 | 0.001 | 0.003 | 0.006 | -0.024 | 1264.0 | 1591.0 | 2256.0 | 1.010 | 3583.0 |
| MEAN | | -0.039 | 0.007 | 0.073 | 0.018 | 0.050 | 0.032 | 0.0245 | 180.743 | 682.416 | 2257.637 | 1473.848 | 1368.502 | 1340.372 |
| MEDIAN | | -0.014 | 0.002 | 0.006 | 0.022 | 0.013 | 0.004 | 0.007 | 0.111 | 0.587 | 2256.5 | 2112.0 | 1247.0 | 1338.0 |

Table S3: IDEXX *M. bovis* antibody ELISA sensitivity to positivity ratios for 21 African buffalo over the study period.

| **Animal Identity** | **Vaccine treatment group** | **MA00** | **MA01** | **MA03** | **MA04** | **MA05** | **MA06** | **MA07** | **MP08** | **MP09** | **MP10** | **MP11** | **MP12** |
| --- | --- | --- | --- | --- | --- | --- | --- | --- | --- | --- | --- | --- | --- |
| 2 | **CONTROL** | 0.084 | 0.075 | 0.006 | 3.304 | 3.328 | 1.004 | 0.207 | 0.274 | 0.740 | 0.171 | 0.120 | -0.003 |
| 15 |  | 0.016 | 0.022 | 0.055 | 0.794 | 3.472 | 0.650 | -0.041 | 0.538 | 1.143 | 0.156 | -0.043 | 0.018 |
| 21 |  | 1.656 | 0.400 | 0.195 | 0.115 | 0.460 | 0.216 | 1.213 | 1.062 | 0.687 | -0.007 | -0.013 | -0.018 |
| 26 |  | 0.001 | 0.032 | 0.251 | 0.805 | 0.483 | 1.000 | -0.046 | 0.365 | 0.353 | 2.685 | -0.110 | -0.020 |
| **MEAN** | | 0.440 | 0.132 | 0.127 | 1.255 | 1.936 | 0.717 | 0.333 | 0.560 | 0.731 | 0.751 | -0.012 | -0.006 |
| **MEDIAN** | | 0.050 | 0.054 | 0.125 | 0.800 | 1.906 | 0.825 | 0.083 | 0.452 | 0.713 | 0.163 | -0.028 | -0.010 |
| 3 | **BCG** | 0.092 | -0.010 | 0.027 | 0.025 | 0.082 | -0.018 | 0.293 | -0.021 | 0.023 | 0.258 | -0.123 | 0.075 |
| 16 |  | 0.010 | -0.028 | 0.032 | 0.180 | 0.401 | 0.504 | -0.082 | 0.087 | -0.040 | 0.122 | 0.010 | 0.350 |
| 22 |  | 0.086 | 0.131 | 0.031 | 0.375 | 0.089 | 0.377 | -0.059 | 1.762 | 0.033 | 1.985 | 1.350 | 0.053 |
| 23 |  | 0.010 | 0.005 | -0.020 | 0.026 | 0.406 | 2.466 | -0.053 | 1.861 | 3.637 | 0.352 | -0.067 | -0.075 |
| 25 |  | 0.205 | 0.050 | 0.242 | -0.021 | 0.247 | 0.139 | -0.089 | 0.311 | -0.037 | 0.215 | 0.150 | 0.010 |
| **MEAN** | | 0.081 | 0.029 | 0.062 | 0.117 | 0.245 | 0.694 | 0.002 | 0.800 | 0.723 | 0.586 | 0.264 | 0.083 |
| **MEDIAN** | | 0.086 | 0.005 | 0.031 | 0.026 | 0.247 | 0.377 | -0.059 | 0.311 | 0.023 | 0.258 | 0.010 | 0.053 |
| 4 | **HIMB-IM** | 0.929 | 0.167 | 0.095 | 1.871 | 1.745 | 1.207 | 14.360 | 2.775 | 0.057 | 1.247 | 0.523 | 0.188 |
| 6 |  | 0.197 | 0.356 | 0.227 | 1.991 | 0.435 | 0.524 | -0.083 | 0.279 | 0.093 | 0.181 | -0.117 | 0.100 |
| 8 |  | 0.014 | 0.005 | -0.009 | 0.095 | 0.235 | 0.084 | 0.005 | 3.684 | 0.103 | 0.849 | -0.067 | -0.043 |
| 10 |  | -0.014 | -0.005 | -0.020 | 0.268 | 0.657 | 0.010 | -0.095 | 0.130 | 0.020 | -0.059 | 0.227 | 0.273 |
| 14 |  | -0.013 | -0.017 | -0.006 | -0.006 | -0.024 | 0.024 | -0.086 | 0.119 | 0.037 | 0.378 | -0.107 | 0.273 |
| 17 |  | 0.059 | 0.005 | -0.025 | 0.028 | 0.229 | 0.101 | -0.099 | 2.120 | -0.100 | 1.174 | -0.097 | -0.058 |
| **MEAN** | | 0.195 | 0.085 | 0.044 | 0.708 | 0.546 | 0.325 | 2.334 | 1.518 | 0.035 | 0.628 | 0.061 | 0.122 |
| **MEDIAN** | | 0.036 | 0.005 | -0.007 | 0.181 | 0.335 | 0.092 | -0.085 | 1.200 | 0.047 | 0.613 | -0.082 | 0.144 |
| 1 | **HIMBO** | 0.080 | -0.016 | 0.028 | 0.262 | 0.114 | -0.020 | 10.783 | 0.343 | 0.217 | 0.482 | -0.127 | 0.120 |
| 5 |  | 0.076 | 0.066 | -0.007 | 1.650 | 0.926 | 0.485 | 14.760 | 1.194 | 0.680 | 0.553 | 0.083 | 4.860 |
| 7 |  | 0.022 | -0.003 | 0.008 | 0.122 | -0.011 | 0.054 | 0.077 | 0.009 | 0.417 | 0.085 | -0.080 | -0.045 |
| 18 |  | 0.065 | 0.001 | -0.007 | 0.018 | 0.098 | 0.043 | 0.148 | 0.864 | 0.087 | -0.022 | 2.587 | -0.090 |
| 20 |  | 0.100 | 0.042 | 0.088 | 0.559 | 0.052 | 0.032 | -0.059 | 0.775 | 0.090 | 0.393 | -0.080 | 0.008 |
| 24 |  | 0.141 | 0.313 | 0.144 | 3.678 | 0.176 | 0.283 | -0.053 | 1.416 | -0.103 | 2.641 | 0.117 | 0.028 |
| **MEAN** | | 0.081 | 0.067 | 0.042 | 1.048 | 0.226 | 0.146 | 4.276 | 0.767 | 0.231 | 0.688 | 0.417 | 0.813 |
| **MEDIAN** | | 0.078 | 0.022 | 0.018 | 0.411 | 0.106 | 0.049 | 0.112 | 0.819 | 0.153 | 0.437 | 0.002 | 0.018 |

Figure S3: Comparison of Sample-to-positive ratios (S/P) per treatment group, for pre-infection period (MA00 – MA07) and post-infection (MP08 – MA12) periods in 21 African buffalo vaccinated against *Mycobacterium bovis* (solid lines represent medians).
